# Asan Medical Center Plastic Surgery Visit: Insights from France

**DOI:** 10.1055/a-2726-3307

**Published:** 2026-01-20

**Authors:** Elise Lupon

**Affiliations:** 1 University Institute of Locomotor and Sport (IULS), Pasteur Hospital, Nice, France; 2 Université Côte d'Azur, National Centre for Scientific Research (CNRS), Laboratoire de Physiomédecine Moléculaire (LP2M), Nice, France

Asan Medical Center (AMC) in Seoul is widely regarded as one of the leading institutions in plastic and reconstructive surgery worldwide. Its reputation for excellence, high surgical volume, and cutting-edge research attracts visitors from across the globe. Following the recent reflections, 1 who provided a detailed description of the department's organization and dynamics, complementary insights may be offered from a European perspective after a 2-week observership at the AMC.

The department is characterized by both organizational efficiency and a healthy leadership structure. On a daily basis, fellows play a pivotal role in coordinating surgical schedules and ensuring a smooth interface between residents and faculty. At the institutional level, leadership responsibilities are passed on regularly, with successive chairs taking over after a few years in a structured and deliberate manner. This model provides continuity while allowing fresh perspectives, fostering a dynamic academic environment that prevents the rigidity sometimes observed in more static systems.

The diversity and complexity of the surgical cases observed within a short period were noteworthy. From complex microsurgical reconstructions to refinements in perforator flap harvest, the exposure provided a concentrated overview of contemporary reconstructive practice at the highest level. 2 3 A particularly memorable highlight was the opportunity to attend a lecture by Professor Cederna on advanced prosthetics, exemplifying AMC's role as a hub for international collaboration. Such events underscore the center's openness not only to visiting observers but also to external expertise, reinforcing its status as a truly global institution.

Equally impressive is the leadership style demonstrated by Professor Joon Pio Hong and his colleagues. Diplomacy, accessibility, and humility characterize an environment in which international observers are not passive spectators but are fully integrated into academic activities. This inclusive ethos fosters a sense of belonging rarely achieved in short observerships and reflects a philosophy of leadership rooted not only in technical mastery but also in mentorship and the cultivation of future leaders. 4 5

The degree of inclusion extended to visiting surgeons is noteworthy. Observers are integrated into ward rounds, operating room discussions, and academic conferences from the outset. This openness extends beyond the hospital walls, facilitating cultural immersion and personal connections. 6 Such dimensions, often overlooked in formal reports, are critical in shaping the overall educational impact: Technical skills are honed in the operating room, but professional identity is strengthened through collaboration and cultural exchange.

For European trainees, the AMC model provides valuable lessons. Structured leadership, inclusiveness, and responsiveness were identified as key elements supporting both clinical excellence and academic productivity. The AMC observership provided additional motivation to advance initiatives already envisioned by French leaders in reconstructive surgery. 7 In this context, ongoing efforts gained new momentum and ultimately led to the establishment of an annual perforator flap training course for surgical residents in France. This illustrates the ripple effect of international observerships, with benefits extending far beyond the hosting institution.

Even a brief observership at AMC offers important lessons in surgical technique, organizational efficiency, and leadership. It exemplifies how international exchange can strengthen both individual careers and broader educational ecosystems in the visitors' home countries. In France, the visit contributed to renewed momentum for educational initiatives, underscoring the value of international collaboration. As Isaac Newton famously stated, “If I have seen further, it is by standing on the shoulders of giants.” The AMC experience stands as a testament to this principle, combining technical excellence with humility and inclusiveness, and inspiring new directions in surgical education abroad.
